# Neurofeedback in children with attention-deficit/hyperactivity disorder (ADHD) – a controlled multicenter study of a non-pharmacological treatment approach

**DOI:** 10.1186/1471-2431-14-202

**Published:** 2014-08-13

**Authors:** Martin Holtmann, Benjamin Pniewski, Daniel Wachtlin, Sonja Wörz, Ute Strehl

**Affiliations:** 1 Hospital for Child and Adolescent Psychiatry, LWL University Hospital Hamm of the Ruhr-University Bochum, Heithofer Allee 64, 59071 Hamm, Germany; 2 Interdisciplinary Centre for Clinical Trials (German abb.: IZKS), University Medical Centre Mainz, Langenbeckstr. 255131 Mainz, Germany; 3 Institute of Medical Psychology and Behavioral Neurobiology, Eberhard-Karls-University Tübingen, Silcherstr. 5, 72076 Tübingen, Germany

**Keywords:** Neurofeedback, SCP, Slow cortical potentials, ADHD, Attention-deficit/hyperactivity disorder, Electromyogram, EMG biofeedback

## Abstract

**Background:**

Attention-deficit/hyperactivity disorder (ADHD) is the most common neurobehavioral disorder of childhood and has often a chronic course persisting into adulthood. However, up to 30% of children treated with stimulants either fail to show an improvement or suffer adverse side effects, including decreased appetite, insomnia and irritability and there is no evidence of long term efficacy of stimulants for ADHD. A series of studies has shown that neurofeedback is an effective additional or alternative treatment for children with ADHD, leading to e.g. significant and stable improvement in behavior, attention and IQ. Significant treatment effects of neurofeedback have also been verified in meta-analyses. Most of the trials, however, have been criticized for methodological difficulties, particularly lacking appropriate control conditions and number of patients included. This randomized study examines the efficacy of slow cortical potentials (SCP) -neurofeedback, controlling unspecific effects of the setting by comparing two active treatment modalities.

**Methods/Design:**

A total of 144 patients with ADHD, older than six and younger than ten years, in some cases with additional pharmacological treatment, are included in this trial. In five trial centres patients are treated either with SCP-feedback or electromyographic (EMG) -feedback in 25 sessions within 3 months. A comprehensive test battery is conducted before and after treatment and at follow-up 6 month later, to assess core symptoms of ADHD, general psychopathology, attentional performance, comorbid symptoms, intelligence, quality of life and cortical arousal.

**Discussion:**

The efficacy of SCP-feedback training for children with ADHD is evaluated in this randomized controlled study. In addition to behavior ratings and psychometric tests neurophysiological parameters serve as dependent variables. Further, the choice of EMG-biofeedback as an active control condition is debated.

**Trials registration:**

Current Controlled Trials ISRCTN76187185. Registered 5 February 2009.

## Background

Attention-deficit/hyperactivity disorder (ADHD) is the most common neurobehavioral disorder of childhood with an estimated prevalence of 3-5% in school-aged children [1,2]. Core symptoms include according to DSM-5 impaired attention, excessive motor activity, and impulsivity [3]. The disorder often has a chronic course with 30-65% of affected children displaying ADHD symptoms in adulthood [4]. Numerous problems are associated with ADHD, including poor social relationships, higher risk taking behavior, and a higher incidence of anxiety and depression symptoms. Stimulant medication, e.g. methylphenidate, represents the most commonly used intervention for children with ADHD. However, up to 30% of children treated with stimulants either fail to show an improvement or suffer adverse side effects, including decreased appetite, insomnia and irritability as well there is no evidence of long term efficacy of stimulants for ADHD [5,6]. After medication washout children experience considerable loss of improvement [7]. Kratochvil et al. [8] reported on the effectiveness and tolerability of long-term atomoxetine treatment. During a period of 2 years, more than 25% of young children with ADHD discontinued the treatment because of lack of effectiveness.

Neurofeedback (NF) as an additional or alternative treatment is based on neurophysiological changes characteristic of ADHD children [9-11] (for an overview, see Becker & Holtmann, [12]; Holtmann & Stadler [13]). This intervention has gained promising empirical support in recent years. Before the initial application of this clinical study, pilot studies had been carried out in three of the five participating centers (Frankfurt, Göttingen, Tübingen). For a group of nearly 50 ADHD children, significant improvement in behavior, attention and IQ was observed after NF. All changes were stable or even improved at six month [14,15] and two year follow-up [16]. Preliminary results of a prospective randomized pilot study in 34 ADHD children comparing NF and a computerized cognitive training indicate that only NF showed specific effects on impulse control [17]. Also, data of a study in children with ADHD comparing NF with a computerized cognitive training found an advantage of NF on behavioral and neurophysiological parameters (Gevensleben et al., 2007). This supports the data of Heinrich et al. [18] who provided first evidence for specific neurophysiological effects of NF in children with ADHD, evidenced in a normalization of the contingent negative variation (CNV), an event-related correlate of attentional resources. In a controlled functional magnetic resonance imaging (fMRI) study, Lévesque et al. [19] demonstrated the capacity of NF to normalize key neural substrates of selective attention in ADHD. Further, it has been demonstrated that NF can lead to microstructural changes in white and gray matter [20].

Several meta-analyses have been published on the effects of neurofeedback on ADHD symptoms. A first meta-analysis examined 467 subjects from 10 prospective, controlled neurofeedback trials [21] and showed medium to high effects for all three core domain of ADHD symptoms. A second meta-analysis, using a more rigorous methodological approach [22] found a significant treatment effect of neurofeedback (ES = 0.59; 95% CL: 0.31-0.87) using ADHD scores from raters (often unblinded) close to the therapeutic setting. Since blinded assessments were only available from four out of the eight included studies, the authors concluded that better evidence for efficacy from blinded assessments is required before neurofeedback can be supported as treatment for ADHD core symptoms. This need is addressed in the present study that applies assessments by a blinded clinical investigator. In addition, there is still lack of evidence whether the observed effects are specific results of the neurofeedback treatment and are not triggered by unspecific effects.

Many studies on neurofeedback in ADHD have been criticized for lacking appropriate controls and follow-up, failing to randomly allocate participants to treatment conditions, using poor diagnostic criteria, and employing subjective and unblinded outcome measures [13,23]. In addition, they failed to take into account the influence of the training setting provided by extensive biofeedback. These limitations inhibit the acceptance of neurofeedback within the psychiatric and psychological communities. Therefore, the aim of this randomized, controlled trial with parallel groups is to examine the efficacy of neurofeedback in comparison to a self-regulation training using a peripheral physiological parameter, controlling for possible unspecific positive effects of the training setting. First, we hypothesize that neurofeedback will show a beneficial effect on the core symptoms of children with ADHD that is superior compared to the control condition. Second, we anticipate that patients will be able to sustain clinical improvement following neurofeedback after having washed out pharmacotherapy. Furthermore, we want to investigate the effect of NF on the degree of illness, the attentional performance, comorbid symptoms, intelligence, quality of life and cortical arousal. For the first time adverse events as well as severe adverse events will be assessed.

## Methods

### Patricipants and recruitment

Participants are being recruited and treated in the five trial centers (LWL University-Hospital Hamm, Ruhr-University Bochum; Central Institute of Mental Health Mannheim; Institute of Medical Psychology and Behavioral Neurobiology, Eberhard-Karls-University Tübingen; Department of Child and Adolescent Psychiatry, University of Göttingen; and Department of Child and Adolescent Psychiatry and Psychotherapy, Goethe-University Frankfurt) as outpatients from seven to under 10 years with an ADHD diagnosis (for all eligibility criteria see Table 1). The diagnosis is being verified in a semi structured interview based on the German adaptation of the Kiddie-Sads-Present and Lifetime Version (K-SADS-PL [24]) by one of the main investigators. Considering the sample size calculation, 144 subjects are enrolled in the clinical trial (72 subjects per treatment group). The Interdisciplinary Center for Clinical Trials (IZKS) of the University of Mainz is responsible for monitoring the study and for project-, safety-, data management and biostatistics. The IZKS Mainz is supported by the grant “Clinical Trial Centers [Klinische Studienzentren], no. FK 01KN1103, IZKS Mainz” from the Federal Ministry of Education and Research.

**Table 1:** Eligibility criteria

	
Inclusion criteria	Attention-deficit/hyperactivity disorder (combined type) (DSM-IV)
	Being 7 to 9 years of age
	Ability to understand character and individual consequences of the trial
	Written informed consent of the person with primary custody must be available before enrolment in the trial
Exclusion criteria	Diagnosis of bipolar disorder, psychosis, serious OCD, chronic serious tics or Tourette syndrome
	Major neurological or medical illness
	Pharmacotherapy for severe anxiety and mood disorders and psychosis
	Acute suicidal tendencies
	IQ below 80 (CPM)
	Non-German speaking child and primary caretaker
	No telephone
	Pregnancy and lactation
	Participation in other clinical trials and observation period of competing trials, respectively.

*CPM* Coloured Progressive Matrices, *DSM-IV* Diagnostic and Statistical Manual of Mental Disorders, version IV, *OCD* Obsessive Compulsive Disorder.

### Ethics and written consent

This study (ISRCTN76187185) was approved by all local Ethics Committees according to the Declaration of Helsinki. Before entering the study patients are informed about the study objectives, study design, and potential risks by one of the main investigators and receive this information in writing also. Written consent is obtained from all participants and their persons in charge of primary custody. In addition children sign an adequate informed consent.

### Interventions

#### *Experimental group: feedback of slow cortical potentials*

Interventions were conducted following protocols of previous controlled studies with positive neurofeedback outcomes [25-28] (for an overview, see Gevensleben et al. [29]; Arns et al. [30]]). Slow cortical potentials (SCPs) are very slow changes in the EEG and belong to the family of event related potentials. They reflect the excitability of the underlying cortical area. While negative shifts mobilize resources for the preparation of motor and cognitive answers to a stimulus, positive shifts reflect inhibition of mobilization [15].

Participants randomly allocated to the experimental group receive 25 sessions of neurofeedback of SCPs, with each session lasting about 60 minutes. This includes time needed for electrode montage as well as four 10 minutes blocks of feedback. Each block consists of 40 trials and each trial is composed of a baseline-phase (2 seconds) and a feedback-phase (8 seconds). The feedback electrode is being placed at Cz and referenced against an electrode behind the left ear (mastoid). In addition one ground electrode is placed on the right mastoid and four electrodes around the eyes to enable an online correction of artefacts produced by eye movements. The training protocol prompts either negative potential or positive potential shifts compared to the baseline [15]. If patients are successful for at least 2 seconds in total during the second half of the feedback-phase a sun is shown as a positive reinforcement. Negativation and positivation are trained in a randomized succession, within the first 12 training sessions in a 1:1 ratio. Respectively the focus on negativation, the ratio is being changed to 4:1 within the last 13 sessions.

Participants are sitting in front of a computer screen and connected to a multichannel amplifier which records EEG as well as EMG activity (NEURO PRAX®; neuroConn GmbH, Ilmenau, Germany). Using previous research as a basis the treatment is being administered two to three times a week, and feedback animations provided are simple. During each session feedback is given in block 1, 2 and 4. To enable transfer of self-regulation skills to everyday life participants perform the third block of each session without continuous feedback (transfer trials). Between the 12th session and the 13th session a period of 3 weeks without training sessions is being arranged. During that period participants have to conduct short transfer exercises where they use the strategies of self-regulation at least three times a day. Little memo-cards that depict the monitor with a successful self-regulation trial serve as a cue to remind the participants to exercise the self-regulation. In addition they receive a DVD with a video showing trials with both tasks simulating the situation in the lab to prompt self-regulation at home. To strengthen the transfer the last 10 sessions are followed by a short transfer exercise as described above before doing some homework in the lab. Participants are able to earn a certain amount of tokens for taking part and good cooperation. These tokens are honored by little presents or vouchers as soon as a certain amount is collected.

#### *Control group: feedback of electromyographic activity of the musculi supraspinatus*

**Figure 1:**
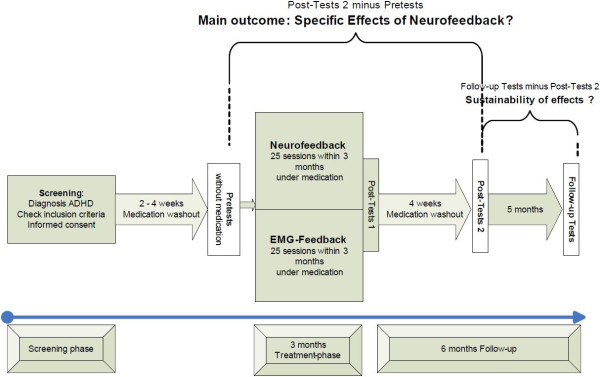
Flow chart.

To control the degree of researcher-participant interaction, as well as other non-specific effects of the feedback-setting, children assigned to the control group attend 25 sessions of electromyography (EMG-) biofeedback. The training protocol reinforces muscular contraction and relaxation of either the left in relation to the right Musculus supraspinatus or vice versa, compared to the baseline. Two electrodes are placed at the upper shoulder area (left and right). Participants of the control group are being instructed to move the object on the screen by contracting and relaxing these muscles. Duration of sessions, amount of transfer trials, surface of the feedback monitor, reinforcement schedules, electrode montage and transfer exercises are identical in both groups.Both interventions are being administered in addition to treatment as usual (TAU). TAU may comprise pharmacotherapy with psychostimulants (short or long acting), e.g. methylphenidate and amphetamine salts, and atomoxetine, and medication for Oppositional Defiant Disorder (ODD) and Conduct Disorder (CD), that are treated pharmacologically similar to ADHD. To assess the unique effects of the interventions, their stability over time and the need of further pharmacotherapy, medication is being washed out before the pretest to establish an unmedicated baseline and after the intervention phase in both groups. The duration of the washout is adapted to the type of medication, with a 2-week period for psychostimulants and 4 weeks for atomoxetine. In cases of lacking practicability a minimum washout period of 48 hours (for psychostimulants) and/or 5 days (for atomoxetine) is allowed and medication is administered again in case of relapse. The timeline of the study is shown in Figure 1.

#### *Randomization and blinding*

After screening, patients are randomly assigned to either the neurofeedback training (experimental group) or electromyographic (EMG)-biofeedback training (control group). Randomization is stratified according to trial site and sex in a 1:1 ratio (neurofeedback vs. EMG-biofeedback). Other prognostic factors are considered by means of adjusted analysis. A web based randomization tool developed at IZKS Mainz is used within this trial. Randomization lists generated at IZKS Mainz by means of a SAS (Statistical Analysis System) program are imported into this tool. For practical reasons, the primary investigators of the study who deliver treatments (but are not involved in outcome ratings) are aware of the participants’ allocation, but all medical consultants are blinded to the allocation. Also parents and participants are not being informed about the randomization outcome but from the start of the training sessions the latter are receiving instructions dependent on the outcome. All participants are connected with both peripheral (EMG) and scalp (EEG) electrodes. Training devices and software are identical for both interventions.

### Assessments

#### *FBB-ADHS*

The German ADHD Rating scale consists of 20 items that assess the severity and perceived burden of inattention, hyperactivity, and impulsiveness as defined by the ICD-10 and DSM-IV and has been used widely in treatment studies for ADHD [31]. It can be used to directly compare effect sizes between different kinds of interventions. The German version has shown good psychometric properties, with good internal consistency (*α* coefficient = 0.78 – 0.89) for parent-rating [32] and (*α* coefficient > 0.90) for teacher-rating, and good inter-rater reliability (r = 0.70) [33].

### Clinical Global Impression Scale

Assessment of general psychopathology is being performed using the Clinical Global Impression Scale (CGI) to estimate symptom severity (CGI-S) and improvement (CGI-I) [34,35]. The CGI is a seven-point scale that requires a rating of illness severity at the time of assessment. Because severity estimation in the CGI is performed in relation to other patients, it is a subjective assessment tool. Ratings are performed by a medical consultant blinded to group allocation.

### Testbattery for Attentional Performance

The Testbattery for Attentional Performance (TAP) examines a large spectrum of specific attentional performances in a computerized form [36]. The following two subtests are being administered.

1) Go/Nogo. This subtest assesses the ability to suppress a response in the presence of irrelevant stimuli. Omission errors, committed errors, average reaction time and intraindividual variance of reaction time are analyzed in both subtests to estimate changes in the attention performance.

2) Flexibility (non-verbal). This subtest assesses the ability to shift the attentional focus, measuring reaction times for valid and invalid cues in a visual task.

The TAP is a well validated measurement with satisfying psychometric properties [36].

### Neurophysiological parameters: a) quantitative EEG and b) event related potentials

Two neurophysiological parameters are ascertained using an EEG amplifier (19 electrodes, 10–20 system):

a) Quantitative EEG (QEEG) is recorded during 6 minutes resting condition (3 minutes eyes closed and 3 minutes eyes open) and during the first, last and follow-up training session, with the same equipment used for feedback but at 19 electrodes. After correction for eye artefacts from the recording channels power spectral analysis, displayed as a topographical brainmap showing the absolute and relative power for delta, theta, alpha and beta frequency are analyzed.

b) Event related potentials (ERPs) are recorded in a cued Continuous Performance Task (CPT-OX [37]). The CPT-OX measures a person's sustained and selective attention and impulsivity. By its embedded Go/Nogo task the CPT-OX captures a sequence of attentional and preparatory brain processes initiated by the cue stimulus (indexed by Cue P300 and CNV) which precede inhibitory control (indexed by Nogo P300 (e.g. [38]). The average reaction time, number of omission and commissions and mean amplitude of CNV and P300 is assessed. Additionally Error-related negativity (ERN, a marker of action monitoring and error processing) is recorded in an Erikson flanker task (ERN-FT [39]). Average reaction time and number of errors for each condition (congruent/incongruent) is assessed.

### Child Behavior Checklist

The Child Behavior Checklist (CBCL) is one of the best-studied, empirically derived parent checklists for measuring general child and adolescent psychopathology [40] and is applied to assess comorbid symptoms. The child’s behavior over the past 6 month using a total of 118 items (plus 2 optional questions) is rated by parents or primary caregivers. The questionnaire includes a total problem score, two superior scales (externalizing problems and internalizing problems), and eight syndrome scales (withdrawal, somatic complaints, anxiousness/depression, social problems, thought problems, attention problems, delinquent behavior, and aggressive behavior). The reliability, factorial validity and discriminant validity of the German adaptation of the CBCL have been confirmed by several studies [41,42]. In case of comorbid symptoms (assessed by the individual syndrome scores) additional corresponding parents’ rating scales of the Diagnostic-System for Mental Disorders in Children and Adolescents (DISYPS-KP [43]) are applied.

### Strengths and Difficulties Questionnaire

In addition to the CBCL, the Strengths and Difficulties Questionnaire (SDQ) is used in order to assess short-term changes of comorbid symptoms and evaluate parent and teacher ratings. The SDQ is a brief behavioral screening questionnaire assessing 25 attributes, some positive and others negative, which can be allocated to five scales (emotional symptoms, conduct problems, hyperactivity/inattention, peer relationship problems, and pro-social behavior) [44]. These scales can be summed to calculate a total difficulties score with the advantage of being able to assess short-term changes [45]. In this study parent and teacher ratings with the SDQ are used. The sensitivity to change of SDQ allows estimation of treatment efficacy (*α* = 0.73; retest stability = 0.62) [46] and monitoring of symptom changes compared to screening.

### Coloured Progressive Matrices

Full scale intelligence quotient (IQ) is measured using the Coloured Progressive Matrices (CPM) [47]. The CPM is a language-free intelligence screening test consisting of 36 items and standardized for children between 4 and 11 years of age. It provides a parallel version (Split-Half Reliability of r = 0.85 to 0.90) to minimize test-retest-effects (test-retest coefficient r = 0.86 to 0.90).

### Quality of life questionnaire

The revised German Kid-KINDL(R) quality of life 24 item questionnaire is a reliable, valid and practicable instrument [48] yielding six dimensions (body, psyche, self-esteem, family, friends, and functional aspects) and a total score. The self-rating for children between 7 and 13 years of age is applied to participants. The internal consistency for subscales reached values from *α* = 0.54 to *α* = 0.73, with an *α* = 0.82 for the total score [49].

### Parents’ expectations on-/satisfaction with therapy

The expectations before treatment, and the satisfaction with therapy during and after treatment is rated by parents using a questionnaire that was developed by the Institute of Medical Psychology and Behavioral Neurobiology, Tübingen [50]. The questionnaire consists of six questions based on a 6-point Likert scale. To avoid bias through social expectancy parents submit the questionnaire directly to IZKS Mainz.

### Adverse events and serious adverse events

At each contact (assessment and training-session) participants are asked to report any adverse events (AEs). AEs are assessed by using open questions, asking about general AEs and their severity during the study period.

#### *Time points of assessments*

The assessment time points are as follows.

• Screening: Eligibility criteria are examined in a first appointment.

• Pre-test: After washing out the medication as outlined above the initial assessment is carried out to establish an unmedicated baseline.

• Post-test I: After 3 months of either neurofeedback or EMG-biofeedback, there is a comprehensive post-therapy assessment.

• Post-test II: One month later, a second comprehensive post-therapy assessment is provided. To compare an unmedicated state to the baseline (established in the Pre-test) again medication is washed out before.

• Follow-up-test: Six months after the training-phase has ended, another comprehensive assessment is carried out. Medication is washed out before.

Additionally the parent version of the FBB-ADHS is assessed monthly. An overview of the time points and the applied assessments is given in Table 2. All questionnaires are presented in German, and are being completed within the different treatment centers with exception of the teachers’ assessments (surface mail) and the monthly assessment of the FBB-ADHS after Post-test II (phone).

**Table 2:** Time points of assessments

**Visit**	**Screening**	**Pre-test**	**Treatment Phase**	**Post-test I**	**Post-test II**	**Follow-up**	**Follow-up-test**
**Action**							
**Trial month**	**-1**	**0**	**1 to 3**	**End of 3**	**End of 4**	**5 to 9**	**End of 9**
FBB-ADHS (Parents)	X	X	XX	X	X	XXXX	X
FBB-ADHS (Teacher)		X			X		X
CGI-S /I	X	X		X	X		X
TAP		X		X	X		X
QEEG, ERPs		X		X	X		X
SDQ (Parents)		X		X			X
SDQ (Teacher)		X					
CPM /parallel	X	X			X		
Kid-KINDL(R)		X		X	X		X
Adverse events		Continuously					
Parents’ expectations/satisfaction	X	X		X	X		X
EFB-K	X						
CBCL	X						

*CBCL* Child Behavior Checklist, *CGI* Clinical Global Impression Scale-Severity /Improvement, *CPM* Coloured Progressive Matrices /parallel version, *EFB-K* Erziehungsfragebogen (German version of the PS, Parenting Scale), *ERPs* Event related potentials, *FBB-ADHS* Fremdbeurteilungsbogen für Aufmerksamkeitsdefizit-/Hyperaktivitätsstörungen (German ADHD rating scale), *Kid-KINDL(R)* Revised German quality of life questionnaire, *QEEG* Quantitative EEG, *SDQ* Strengths and Difficulties Questionnaire, *TAP* Testbattery for Attentional Performance.

### Primary and secondary endpoints

The primary endpoint of this study is the change in ADHD rating scale (FBB-ADHS) after treatment and washout of medication (Post-test II minus Pretest).

Secondary endpoints include:

• CGI-I.

• Resumption of medication by choice of family during follow-up.

• Change in neuropsychological and neurophysiological parameters.

• Change in SDQ questionnaire subscales.

• Change of full-scale IQ in CMP.

• Change in KINDL(R) questionnaire (both parents and child version).

• Score measuring parents' satisfaction with therapy.

### Statistics

#### *Pre-specification*

A detailed methodology for summary and statistical analysis of the data collected in this trial is documented in a Statistical Analysis Plan (SAP) that is dated and maintained by IZKS Mainz. The document may modify the plans outlined in the study protocol; however any major modifications of the primary endpoint definition and/or its analysis are also reflected in a protocol amendment. The SAP has to be authorized before database closure by the biometrician and the coordinating investigator.

#### *Sample-size calculation*

Estimates of a clinically relevant effect size were derived from the Göttingen pilot-study using the same primary outcome measures [18]. It is expected that in the neurofeedback group the mean FBB-ADHS score at Post-Test 2 is 1.20 and in the control group 1.50 with a common standard deviation of 0.55. The expected outcome requires a sample size of 72 subjects per group (*α* = 0.05, two sample t-test, two-sided) to achieve a power of 90%.

#### *Statistical analysis*

Data are analyzed primarily in the modified intention-to-treat (mITT) population. Supportive analyses are planned in the per-protocol (PP) population. mITT comprises all randomized patients with the exception of patients for which it is obvious at the time of randomization that no study specific therapy would be applied, while PP analysis assesses mITT patients who do not meet any of the following criteria: violation of inclusion and/or exclusion criteria, major deviations from the visit schedule, and bad compliance during feedback sessions. All safety parameters are analyzed in the safety population comprising all patients participating in at least one feedback session. Within these analyses patients are analyzed according to the received treatment even if the respective patient was randomized to the other treatment group.

In the primary analysis the primary outcome is tested by an analysis of covariance (ANCOVA) using treatment, trial site, baseline FBB-ADHS score, baseline ADHD medication, parenting style, parent’s expectations, and sex as covariates. The analysis is repeated for the PP population as a sensitivity analysis. In further analyses other potential predictors of response to treatment are examined. Secondary outcome measures comprise binary variables and scores derived from standardized questionnaires. For binary variables proportions and relative risks together with their associated 95% confidence-intervals are calculated. Differences between intervention groups are assessed using logistic regression models. Scores are described by distributional parameters (mean, standard deviation, median, quartiles, and range). In case the assumption of normally distributed data can not be rejected, differences in the scores between intervention groups are analyzed by an ANCOVA using the same predictors as in the primary analysis. In case of major deviations from ANCOVA requirements, appropriate non-parametric methods are applied.

### Quality assurance

#### *Data management*

A detailed methodology for the data management in this trial is documented in a data management plan that is dated and maintained by IZKS Mainz. This plan is signed by the sponsor, the head of the data management team and the responsible data manager. This trial is performed using an electronic case report form (eCRF) or remote data entry (RDE). The investigator and the trial site staff receive system documentation, training and support for the use of the eCRF. During data entry integrity checks help to minimize entry failures. These data entry checks are based on the data validation plan. Any missing data or inconsistencies are reported back to the respective site and clarified by the responsible investigator. After completion of data entry and if no further corrections are to be made in the database, the access rights are taken away and the database is declared closed and used for statistical analysis.

#### *Monitoring*

Monitoring is done by personal visits from a clinical monitor according to prior defined standard operation procedures of the IZKS Mainz. By frequent communications (letters, telephone, fax), the site monitor ensures that the study is conducted according to the protocol and regulatory requirements. The investigator has to allow the monitor to look at all essential documents and has to provide support at all times to the monitor. Furthermore, queries are resolved in cooperation with the investigator. Close-out visits are conducted to close the study site at the end of the study and to ensure that all study-related documents archived.

#### *Advisory board*

An independent advisory board is established. This advisory board is supposed to act as a data monitoring and safety board during non-public meetings in the absence of the principal investigator. The advisory board supervises the conduct of the study and issues recommendations for early termination, modifications or continuation of the study, if necessary. It has to be informed contemporary of serious study related events.

## Discussion

This paper presents the design and protocol of a randomized controlled trial (RCT) with neurofeedback for children with ADHD in an outpatient setting. The choice of EMG biofeedback as a control condition was made after an extensive discussion. Although there is no doubt that double-blind, placebo-controlled trials could provide strong evidence for the efficacy and specificity of a given treatment, there are several issues of a “sham” condition specially in neurofeedback. Apart from the ethical issues, the feasibility of a sham condition for neurofeedback is doubtful. Birbaumer et al. [51] once tried to establish a double-blind sham condition in a neurofeedback trial for patients with epilepsy. Patients as well as the trainer “detected” the sham sessions and refused further cooperation. Furthermore neurofeedback in particular seems to induce the assumption that one is part of the placebo control. In previous placebo-controlled trials of neurofeedback, up to 80% of the participants of the neurofeedback groups estimated (after treatment) that they received placebo feedback [52,53]. As we have learned from one of our pilot studies [15] it takes time until children are able to self-regulate their brain activity. Therefore clinical improvement becomes evident only after a certain delay. The impression of uncontrollability that arises especially in the beginning might assume that missing effects are due to the control condition and therefore may lead patients to discontinue participation. Therefore, the present design made use of EMG biofeedback as an alternative control condition, based on the following considerations: There are not many studies on EMG Feedback available with satisfying methods and/or results. A review of 44 studies [54] concludes that the data do not suggest that biofeedback (i.e. EMG) techniques are superior to more conventional treatment. A detailed analysis of the studies included in this review leads to an even more pessimistic estimation of the effects. Original work published after 1981 does not show much improvement. No study reports follow-up data (e.g. [55]), diagnosis is based on teachers’ ratings [56], some of the improvements are found in the placebo-group as well [57] and EMG Feedback was frequently carried out in addition to other treatments. Arnold [58] concludes from his comprehensive review that EMG Feedback “merits further studies”. Although not tightly connected to the known pathology of ADHD, relaxation of muscles can have effects on the EEG [59]. Therefore possible changes in the EEG are controlled by the QEEG (secondary measure) in this study. As shown by Bakhshayesh et al. [60] EMG Feedback does have an effect on core symptoms of ADHD albeit not as much as EEG-Feedback. Therefore from an ethical viewpoint EMG-Feedback is not just “empty” and senseless, possibly reflecting the desirable placebo effect of the treatment as being rewarded for being attentive, concentrated and cooperative. Moreover it is to mention that a blinded setting might work well in drug research but seems an inappropriate requirement in psychotherapeutic treatments where subjects are supposed to learn a certain behavior. In Biofeedback the task is to acquire a self-regulation skill. At least the first stage of this learning process requires conscious control over the target variable [61]. As observed by Surwit and Keefe [62] without knowing which parameter is being trained subjects are less effective in the acquisition of control. This all amounts to the conclusion that the EMG biofeedback applied in this design is an adequate, satisfying and credible control condition. If the trial supports the effectiveness of neurofeedback in reducing ADHD core symptoms in the absence of stimulant therapy, this would offer an effective alternative for those ADHD patients whose treatment is up to now limited by poor medication response, adverse side effects, and in cases in which the patients and/or their parents refuse medication treatment. In children who respond to pharmacotherapy, medication could be withdrawn after successful neurofeedback training. Beyond the immediate research setting, neurofeedback might be expanded to other behavioral disorders.

The present study examines the effects of neurofeedback on behavioral as well as neurophysiological parameters comprising attentional, preparatory, time processing, and inhibitory ERP components. In a longitudinal study of neuropsychological and electrophysiological markers of different ERP components Doehnert et al. [63] suggest that especially preparatory and time processing brain processes indexed by CNV remained detectable in young adult ADHD subjects, even in ADHD remitters, compared to controls. The results seem to indicate residual timing deficits even in young adults with remitted ADHD. Heinrich et al. [18] investigated the effects of a neurofeedback training aiming at generating a ‘more negative’ CNV in children with ADHD compared with a waiting-list group. Besides a reduction of ADHD symptoms following the training, a pronounced increase of the CNV amplitude was observed in CPT cue trials. The authors suggested that the CNV increase may be interpreted as a neurophysiological correlate of improved self-regulatory capabilities. Expecting a relevant stimulus, children with ADHD may be able to allocate more resources after neurofeedback. Interestingly, similar effects on the CNV were reported following cognitive-behavioral interventions. Against that background future neurofeedback studies may directly aim at the modulation of CNV and explicitly address impaired preparatory processes and timing as an important target of treatment [64].

## Competing interests

This work is funded by the German Research Foundation (DFG; ref: HO 2503/4-1; HO 2503/4-2).

MH has served in an advisory or consultancy role for: Lilly, Novartis, and Bristol-Myers Squibb, and has received conference attendance support or was paid for public speaking by AstraZeneca, Janssen-Cilag, Lilly, Neuroconn, Novartis, Medice, and Shire. BP was paid for public speaking by Lilly and Novartis. US was paid for public speaking by Novartis, Medice, Neuroconn, the German Society for Biofeedback and Akademie König und Müller. The present work is unrelated to the above grants and relationships. The other authors have no conflicts of interest.

## Authors’ contributions

MH and US conceived the research project; they designed the study; and together with SW and DW designed and tailored the study protocol. All authors contributed to the writing of the manuscript. All authors read and approved the final manuscript.

## Authors’ information

Ute Strehl Senior author.

## Pre-publication history

The pre-publication history for this paper can be accessed here:

http://www.biomedcentral.com/1471-2431/14/202/prepub
